# Therapeutic Efficacy of Monoterpenes in Nile Tilapia Infected With *Edwardsiella tarda* : A Phytogenic Alternative to Oxytetracycline

**DOI:** 10.1111/jfd.70032

**Published:** 2025-07-31

**Authors:** Danilo Vitor Vilhena Batista, Adolfo Jatobá, Alexandre Vaz da Silva, Ana Paula de Souza, Caio Francisco Santana Farias, Emilly Monteiro Lopes, Marco Shizuo Owatari, Arlene Sobrinho Ventura, Claudia Andrea Lima Cardoso, Silvia Terra Fontes, Fernando Yugo Yamamoto, Maurício Laterça Martins, José Luiz Pedreira Mouriño

**Affiliations:** ^1^ Aquatic Organism Health Laboratory – AQUOS Federal University of Santa Catarina Florianópolis SC Brazil; ^2^ Aquaculture Laboratory – Laq Federal Institute of Santa Catarina Araquari SC Brazil; ^3^ Laboratory of Algae Cultivation – LCA, Aquaculture Department Federal University of Santa Catarina (UFSC) Florianópolis SC Brazil; ^4^ Faculty of Agricultural Sciences – FCA Federal University of Grande Dourados Dourados MS Brazil; ^5^ Center for Natural Resource Studies – CERNA State University of Mato Grosso Do Sul Dourados MS Brazil; ^6^ Thad Cochran National Warmwater Aquaculture Center, Mississippi Agriculture and Forestry Experiment Station Mississippi State University Stoneville Mississippi USA; ^7^ Department of Wildlife, Fisheries and Aquaculture, College of Forest Resources Mississippi State University Mississippi State Mississippi USA

**Keywords:** aquaculture, carvacrol, phytotherapeutics, thymol, treatment

## Abstract

The purpose of this research was to assess the effectiveness of a combination of monoterpenes (thymol and carvacrol) in treating juvenile Nile tilapia ( *Oreochromis niloticus* ) infected with *Edwardsiella tarda* as a potential substitute for the antibiotic oxytetracycline. The study utilised different concentrations of the monoterpenes blend (MTs): 0.4, 0.6 and 0.8 g kg^−1^ of feed. A negative control group received a diet without the additive, while a positive control group was given medicated feed containing oxytetracycline (OTC) (11.85 g kg^−1^) for a 10‐day treatment period. Cumulative mortality (%), haematological and immunological parameters were assessed after the feed administration. The negative control group experienced a mortality rate of 53%, while fish treated with medicated feed had a significantly lower mortality rate of 3%. The addition of MTs at concentrations of 0.4 g kg^−1^ (13% mortality), 0.6 g kg^−1^ (8% mortality) and 0.8 g kg^−1^ (0% mortality) also resulted in reduced mortality rates, with the highest concentration showing survival rates comparable to the oxytetracycline group. Following the 10‐day treatment period, no mortality was observed in the treated groups, indicating the efficacy of MTs‐supplemented feed in alleviating the clinical symptoms of Edwardsiellosis. The haematological parameters indicated that as the dosage of MTs increased, the mean corpuscular volume (MCV) decreased. The MCV was significantly lower in the 0.6 and 0.8 g kg^−1^ treatments compared to the control. Mean corpuscular haemoglobin initially increased with the 0.4 g kg^−1^ MTs treatment but then decreased with higher doses. The mean corpuscular haemoglobin concentration was higher in the treated groups compared to the control. Red blood cell levels were higher in the 0.8 g kg^−1^ MTs treatment and lower in the 0.4 g kg^−1^ treatment. Ht was higher in the control group and lower in the 0.4 g kg^−1^ MTs treatment. Haemoglobin levels followed a similar trend, with higher values in the control group. The 0.8 g kg^−1^ MTs treatment had the highest number of total leucocytes and lymphocytes. The control group had the highest number of neutrophils, while the 0.8 g kg^−1^ MTs group had higher numbers of monocytes, eosinophils, basophils and thrombocytes. The negative control exhibited the lowest antimicrobial titre compared to the OTC and the various concentrations of MTs. The concentrations of MTs (0.4, 0.6 and 0.8 g kg^−1^) demonstrated similar average values and were more effective than the control and OTC. There was no significant difference in agglutinating titre among MTs, control and OTC. Thymol and carvacrol notably decreased the mortality of tilapia infected with *E. tarda* , with a dosage as high as 0.8 g kg^−1^ showing equivalent effectiveness to oxytetracycline. Furthermore, they enhanced haematological and immunological parameters, indicating that MTs could be a promising alternative to antibiotic use.

## 1 Introduction

The occurrence of bacterial diseases and endoparasites in fish farming systems represents a significant health challenge, especially in aquatic organism breeding farms (Samsing and Barnes 2024). One of the major global bacterial diseases is Edwardsiellosis, a systemic disease caused by *Edwardsiella tarda* (Goh et al. 2023; Maldonado‐Miranda et al. 2022). *E. tarda* is a significant bacterial pathogen in the worldwide fish farming industry, known to trigger disease outbreaks with high mortality rates (Goh et al. 2023; Maldonado‐Miranda et al. 2022; Xu and Zhang 2014). The economic impact of these outbreaks on global aquaculture is estimated to be around US$6 billion annually (Goh et al. 2023). Recent research has shown that in the United States, Edwardsiellosis has caused economic losses of up to US$45.9 million per year in the catfish industry (Kumar et al. 2024). To address this issue, the aquaculture industry continues to rely on the widespread use of antibiotics (Preena et al. 2020).

Misuse of antibiotics in these environments has led to the rise of multidrug‐resistant bacteria, promoting the selection and spread of resistance genes (Preena et al. 2022). Infected fish can act as carriers of antimicrobial resistance, posing a potential threat to human health. Contact with these bacteria, either through consuming inadequately prepared fish or direct exposure, can make treating infections in humans more challenging, as conventional antibiotics may not work against resistant strains (Gazal et al. 2020; Serwecińska 2020). Bacteria acquire resistance through several mechanisms, including inactivation of antibiotics, modification of target structures, or limitation of drug entry into cells (Wilson et al. 2020). Therefore, understanding these mechanisms is crucial for developing effective approaches to address bacterial resistance in aquaculture (Bhat and Altinok 2023; Owatari et al. 2024).

The Food and Agriculture Organisation of the United Nations (FAO) has been documenting the increasing bacterial resistance in farmed fish, recognising its detrimental effects on both aquaculture and public health (FAO 2024; World Health Organization 2021). Recent studies have shown that strains of *E. tarda* found in fish farms exhibit significant resistance to oxytetracycline, amoxicillin and florfenicol (Manzoor et al. 2023), highlighting concerns regarding certain aquaculture practices. This has prompted a global effort to promote more sustainable aquaculture methods, including responsible antibiotic usage and additional research to address the factors contributing to antimicrobial resistance (Bondad‐Reantaso et al. 2023).

In aquaculture, antimicrobial resistance has been observed in Nile tilapia ( *Oreochromis niloticus* ), which is crucial for food security and the global economy (Ashouri et al. 2023; Li and He 2022). The transmission of resistance genes from aquaculture to humans can occur through various pathways, such as consuming contaminated seafood, contact with infected water or soil, and the excessive use of chemicals and antibiotics (Debnath et al. 2023). This can have serious implications for public health, leading to more challenging bacterial infections due to antibiotic resistance (Flores‐Vargas et al. 2024). The spread of these genes among humans can be increased by consuming contaminated seafood, posing a growing challenge for healthcare systems (Sun et al. 2024). Addressing challenges such as bacterial resistance is crucial for the sustainable development of the aquaculture industry, which plays a key role in providing sustainable animal protein to the global population (Elleby et al. 2023; Hasimuna et al. 2020; Li and He 2022).

In recent years, the importance of fish health control, management and nutrition in Nile tilapia farming has been recognised as crucial for sustainable development (Musa et al. 2023). Additional strategies like vaccines, probiotics and feed additives such as phytogenics can be integrated to enhance the immune response of cultured organisms (Jatobá et al. 2016). It is imperative to explore cost‐effective alternative solutions that do not leave residues in fish tissues and effectively combat bacterial infections, considering the risk of bacterial resistance to antimicrobials (Baciu et al. 2024; Tizhe et al. 2024). Given this scenario, phytogenics are seen as powerful substitutes for the indiscriminate use of antibiotics (Abdallah et al. 2023).

Phytotherapeutics have become increasingly popular in aquaculture because of their effective antimicrobial properties and proven efficacy. Furthermore, their natural attributes make them environmentally friendly and less toxic agents (Nikolic et al. 2024). Recent studies have shown that phytogenic compounds can effectively manage Edwardsiellosis in aquaculture, enhance animal growth performance, boost the immune system and increase disease resistance (Goh et al. 2023). For instance, clove basil ( *Ocimum gratissimum* ) and ginger ( *Zingiber officinale* ) essential oils enhanced the immune response and increased the resistance of tilapia to experimental infection by *S. agalactiae* . Furthermore, clove basil essential oil exhibited growth‐promoting effects (Brum et al. 2017). Likewise, an Amazonian phytotherapeutic derived from açai ( *Euterpe oleracea* ) essential oil improved the haemato‐immunological parameters and growth performance of koi carp ( *Cyprinus carpio* ) (Silva et al. 2025). This evidence supports the effectiveness of essential oil in aquaculture.

Among the key organic compounds present in essential oils, thymol and carvacrol, both monoterpenes, are notable. These compounds are naturally occurring in essential oils derived from plants (Kachur and Suntres 2020) and possess antimicrobial and antioxidant properties (Gholami‐Ahangaran et al. 2022). They find applications in various fields, including as food additives and potential therapeutic agents in aquaculture (Frota et al. 2022; Ibrahim et al. 2022; Khalil et al. 2023; Yousefi et al. 2022).

The aim of this research was to assess the effectiveness of incorporating a combination of monoterpenes, thymol and carvacrol into the diet of juvenile Nile tilapia as a postinfection treatment for *E. tarda* , replacing the use of oxytetracycline therapy. This aim is supported by the known antimicrobial, anti‐inflammatory and immunomodulatory properties of monoterpenes (Alvarenga et al. 2023; Zielińska‐Błajet and Feder‐Kubis 2020), making them a promising strategy for managing bacterial infections in aquaculture.

## 2 Material and Methods

The experiment was conducted at the Aquaculture Laboratory (LAq) of the Federal Institute of Santa Catarina (IFC), Araquari Campus. All procedures applied to the animals were approved by the Animal Use Ethics Committee (CEUA/IFC) under the protocol 456/2024.

The lethal dose test and experimental infection were performed using the strain *E. tarda* (ATCC 15947); this strain was isolated from samples of infected fish. This strain is deposited at the Fisheries Institute (São Paulo, SP) and was provided to the strain bank of the Aquatic Organism Health Laboratory—AQUOS/UFSC, where it was used in the experiment in Araquari. The study lasted 22 days, including 2 days for observation of the first clinical signs, 10 days of treatment considering the use of oxytetracycline as a positive control during the experimental period and 10 days of posttreatment observation.

### 2.1 Experimental Animals, Diets and Rearing Conditions

A total of 350 Nile tilapia juveniles (5.0 ± 0.1 g) were obtained from the Empresa de Pesquisa Agropecuária e Extensão Rural de Santa Catarina EPAGRI. For acclimatisation conditions, the fish remained for 7 days in two 250 L experimental units (175 animals per unit) connected to a recirculation system equipped with physical/biological filtration and an ultraviolet (UV) system. During the acclimatisation period, the fish were fed 5% of their biomass with commercial feed (supra juvenile) containing 46% crude protein and a particle size of 1.77 mm (File S1).

After the acclimatisation period, 300 juvenile Nile tilapia were randomly distributed into 20 experimental units (15 fish per unit) connected to a recirculation aquaculture system equipped with physical/biological filtration and an ultraviolet (UV) system. The fish were subjected to five treatments in quadruplicate, labelled as negative control (commercial feed), positive control (feed containing oxytetracycline at a dose of 11.85 g kg^−1^) and three different diets containing monoterpenes at doses of 0.4 g, 0.6 g or 0.8 g kg feed^−1^.

A blend of monoterpenes was supplemented in the feed with the commercial product Digestarom. The commercial product was incorporated into commercial tilapia juvenile feed (46% crude protein; 40% fat and 1.77 mm diameter). The commercial feed was pulverised using a mechanical grinder (TRE25, Tramontina, Carlos Barbosa, RS, Brazil), and gradual levels of Digestarom were supplemented into the mash and homogenised using a concrete mixer (Horbach 150). The resulting mash was moistened with 20% water and cold pelleted (W50, manufacturer, city, country). The pellets were dried at 38°C for 6 h. This protocol was also performed to manufacture the control and the medicated feed supplemented with oxytetracycline (11.85 g). The experimental feeds were packed in airtight bags and stored in a freezer at −20°C. This methodology was adapted according to Silva et al. (2025).

### 2.2 Oxytetracycline and Monoterpenes Analyses

After formulating the experimental diets, the feed was sent for gas chromatography analyses, which was carried out by the Center for Natural Resource Studies, State University of Mato Grosso do Sul (CERNA‐UEMS). The extraction of oxytetracycline to liquid phase was initiated with 1.5 g of homogenised sample and 10 mL of McIlvaine buffer 0.01 mol L^−1^ (pH 4) using an ultrasound. Trichloroacetic acid was added, and the solution was centrifuged (4000 rpm for 5 min). The supernatant was collected and subjected to further extraction. After combining the supernatants, n‐hexane was added and the aqueous phase was cleaned using a polymeric cartridge. The eluate was evaporated (Büchi R‐200 rotary evaporator, Italy), reconstituted in oxalic acid and filtered for HPLC analysis according to Lavorante et al. (2009). The concentration of oxytetracycline in the experimental diet was approximately 9.91 g kg^−1^ of feed.

The analysis of monoterpenes was performed using gas chromatography coupled with mass spectrometry (GC–MS). Each feed sample (5.0 g) was extracted with 30 mL of chromatographic grade hexane in ultrasound for 30 min. The substances were identified using the calculated retention index (RI) employing the linear alkane standard (C7‐C40, Sigma Aldrich with purity ≥ 98%), as well as comparisons of the RI with the indices in the literature (Adams 2007) and interpretation of the mass spectra of the samples and comparison with databases (NIST21 and WILEY229). The results were expressed as a percentage. The results show that monoterpenes were well incorporated into the feed, resulting in a gradual increase in thymol and carvacrol, depending on the amount used.

Thymol was identified as the main monoterpene in the final composition of the feed, with a concentration of 24.8 g kg^−1^. In comparison, carvacrol was quantified in smaller proportions, with 2.9 g kg^−1^, evidencing the predominance of thymol as the main bioactive compound present in the feed. In the analysis of the Digestarom product, other compounds were found in low concentrations, such as methyl salicylate 0.50 g kg^−1^ and e‐anethole 0.10 g kg^−1^. However, these components were not detected in the final composition of the feed using the method employed, with only thymol and carvacrol remaining as bioactive compounds incorporated into the feed.

### 2.3 Lethal Dose 50_96h_ (LD50_96h_ )

The definition of LD50_96h_ consisted of testing different doses of bacterial concentrations in a small number of animals to establish the optimal bacterial concentration to infect the fish, causing clinical signs and mortality of 50% of the population within 96 h. For this purpose, *E. tarda* (ATCC 15947) was used, with a total of 25 fish divided into five experimental units with five fish in each. The animals were anaesthetised with eugenol solution (75 mg L^−1^) and infected with 100 μL of the bacterial solution via gavage, at concentrations of 2 × 10^6^ CFU mL^−1^, 2 × 10^7^ CFU mL^−1^, 2 × 10^8^ CFU mL^−1^ and 2 × 10^9^ CFU mL^−1^, and a control group that received only 100 μL of sterile saline solution (SSE).

### 2.4 Experimental Challenge

For the infection test, 300 animals were anaesthetised with eugenol solution (75 mg L^−1^) according to Libanori et al. (2025) and then infected with 100 μL of a bacterial solution containing the *E. tarda* strain (ATCC 15947) via gavage (Owatari et al. 2022), at a concentration of 2 × 10^7^ CFU mL^−1^ as determined by the LD50. Treatment with the experimental diets against *E. tarda* began when the first clinical signs and/or behavioural changes were observed and continued for 10 days. Animals displaying clinical signs of the disease or that had recently died were used for bacterial reisolation to confirm infection with the experimental pathogen.

During the experiment, water quality variables remained within acceptable conditions for Nile tilapia (Stallbohm et al. 2024). Water quality variables were measured throughout the experimental period and included dissolved oxygen, temperature (YSI PRO20 dissolved oxygen meter), total ammonia nitrogen, total nitrite nitrogen, total nitrate nitrogen and pH (Table 1). All variables were checked three times daily, and the experimental units were siphoned twice a day to remove excess organic solids.

**TABLE 1:** Recirculation aquaculture system water quality parameters (mean ± standard deviation) during the experimental period to evaluate the therapeutic efficacy of monoterpenes in Nile tilapia infected with *Edwardsiella tarda* .

Variables	
Temperature (°C)	25.39 ± 0.09
Dissolved oxygen (mg L^−1^)	7.13 ± 0.42
pH	7.26 ± 0.15
Total ammonia (mg L^−1^)	1.04 ± 0.78
Toxic ammonia (mg L^−1^)	0.003 ± 0.00
Nitrite (mg L^−1^)	0.15 ± 0.05
Nitrate (mg L^−1^)	0.54 ± 0.37

### 2.5 Haematological Analyses

Haematoimmunological analyses were performed only on surviving fish from the experimental units, where they were anaesthetised with eugenol solution (75 mg L^−1^). Blood collection was performed as described by Ranzani‐Paiva et al. (2013), which consists of puncturing the caudal vessel with a 1.0 mL syringe containing an anticoagulant solution, ethylenediaminetetraacetic acid (EDTA 10%).

Blood smears were prepared in duplicate and stained with May‐Grunwald‐Giemsa Wright for total erythrocyte and leucocyte counts and differential leucocyte counts (Ranzani‐Paiva et al. 2013). Another aliquot was used for erythrocyte counting (10^6^ μL^−1^) in a Neubauer chamber (Tavares‐Dias et al. 2002); haematocrit (Ht) percentage was determined by the microhaematocrit method (Goldenfarb et al. 1971) and haemoglobin (Hb) measured using the LABTEST kit, based on the cyanomethaemoglobin methodology, with reading on a biochemical analyser (Thermo Plate), following the recommendations of Collier (1944).

From these data, haematimetric indices were calculated (Vallada 1995): mean corpuscular volume (MCV), mean corpuscular haemoglobin (MCH) and mean corpuscular haemoglobin concentration (MCHC). Total plasma protein (TPP) was determined using a refractometer (Lumen RHC‐200ATC).

The agglutination analysis was performed using 96‐U‐shaped microplates according to Libanori et al. (2025). Samples were serially diluted at 1:2 up to the 12th well. Subsequently, 50 μL of formalin‐inactivated *E. tarda* (10%) was pipetted to all wells. The microplates were then incubated in a humidified chamber at 25°C for 18 h. Agglutination was considered positive when precipitation was observed, and the corresponding dilution was recorded as the reciprocal of the last positive dilution according to Silva et al. (2009). Likewise, the antimicrobial potential of plasma was evaluated against *E. tarda* using 96‐flat‐bottom‐well microplates, adapted from Silva et al. (2009). *E. tarda* inoculum was incubated in BHI broth for 24 h at 30°C and diluted in PB to a final concentration of 2.2 × 10^7^ CFU mL^−1^. Plasma was diluted in PB in a 1:3 ratio in the first well and then serially diluted 1:2 until the 12th well. Saline solution served as positive and negative controls. *E. tarda* was added to the wells with diluted plasma and positive control. After 24 h of incubation at 28°C, bacterial growth was monitored by measuring absorbance at 550 nm. The antimicrobial titre was determined as the highest dilution showing complete inhibition of microbial growth.

### 2.6 Minimum Inhibitory Concentration

To determine the minimum inhibitory concentration (MIC), the *E. tarda* strain (ATCC 15947) isolated from infected fish samples was used. To define the MIC, 100 μL of PB (Poor Broth) culture medium was added to each well of a 96‐well flat‐bottom microplate according to Libanori et al. (2025). In parallel, the same methodology was used to determine the MIC of oxytetracycline (oxytetracycline TM‐700, active ingredient: oxytetracycline hydrochloride, Vetnil, Brazil), using the same culture medium (PB) with a solution of oxytetracycline dissolved in liquid medium at a concentration of 128 μg mL^−1^. The antibiotic was prepared in a sterile solution and added to the wells in serial dilutions. Bacterial suspension was inoculated into each well, and the plate was incubated at 28°C for 24 h. Controls with only culture medium, bacteria and no bacteria were included. Bacterial growth inhibition was observed after 24 h. All tests were done in triplicate.

### 2.7 Statistical Analysis

The homogeneity and normality of the data were assessed using the Levene and Shapiro–Wilk tests, respectively. Nonparametric data were transformed to log_10_. Then, all data were submitted to analysis of variance (one‐way ANOVA). When significant differences were observed (*p* < 0.05), Tukey's test (5%) was applied to compare the means using Statistica v. 12.5 software. Mortality data were submitted to the Kaplan–Meier curve and compared using the log‐rank test. This analysis was performed using GraphPad Prism 10 software.

## 3 Results

### 3.1 Experimental Challenge

**FIGURE 1:**
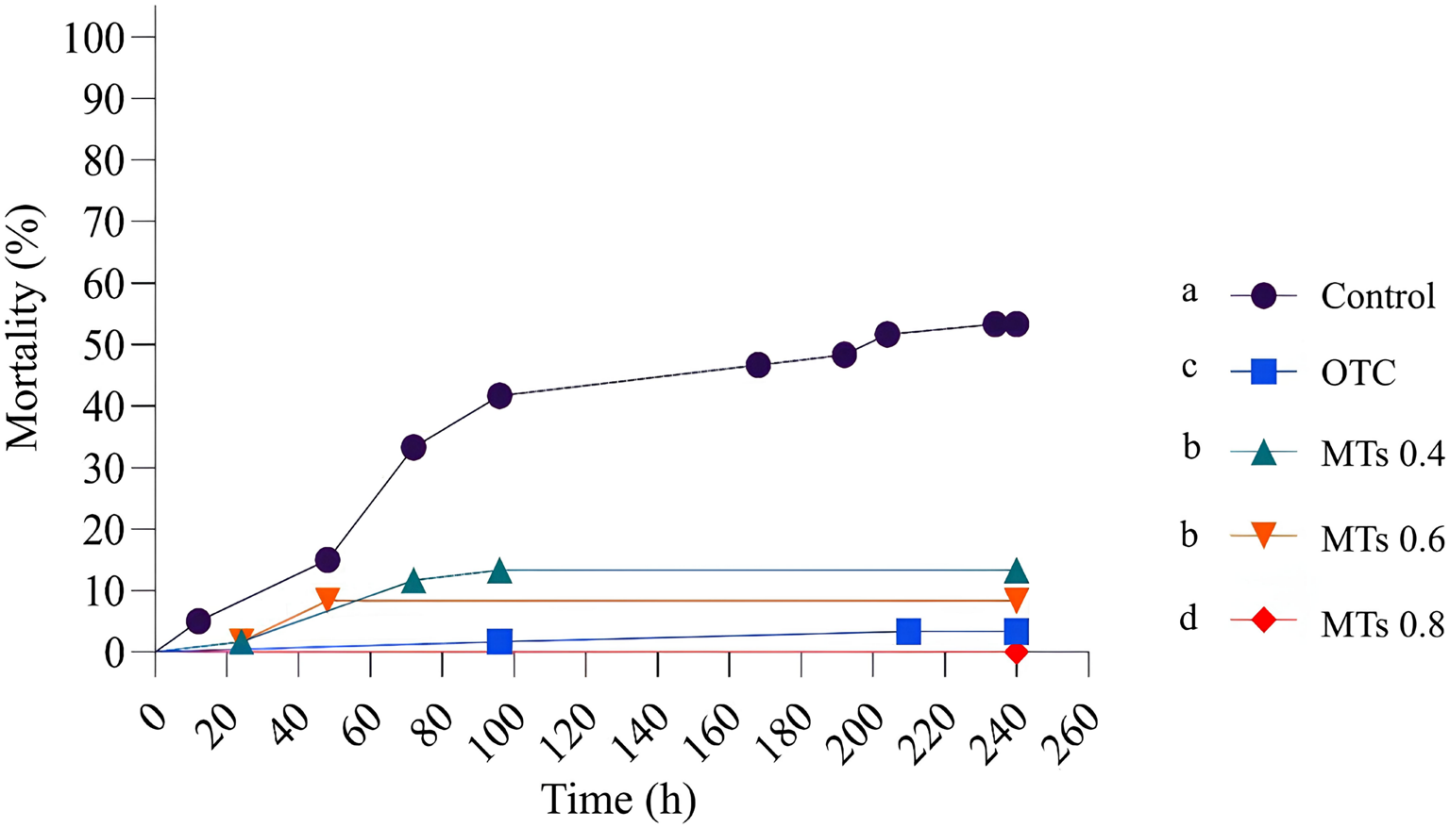
Cumulative mortality of juvenile Nile tilapia ( *Oreochromis niloticus* ) during 240 h (10 days) of treatment with oxytetracycline and different concentrations of monoterpenes in g kg^−1^ of feed.

Fish in the control group experienced increasing mortality, reaching 53% after 240 h (10 days). Fish treated with oxytetracycline (positive control) had a mortality rate of 3%, indicating the effectiveness of the traditional antibiotic. Fish treated with different doses of monoterpenes (MTs) showed lower mortality rates compared to the control group. The 0.4 g kg^−1^ treatment resulted in a mortality rate of 13%, while the 0.6 g kg^−1^ treatment had a mortality rate of 8%, and the 0.8 g kg^−1^ treatment had a mortality rate of 0%, making it the most successful among the treated groups. The mortality control of the 0.8 g kg^−1^ MTs treatment was slightly better than that of oxytetracycline (Figure 1). It is important to highlight that, following the 10‐day treatment period, the surviving fish were monitored for an additional 10 days to assess posttreatment mortality. Throughout this observation period, no mortality was recorded in the groups treated with oxytetracycline and monoterpenes, indicating the disappearance of clinical signs after the treatment duration.

### 3.2 Haematological Analyses

The analysis of haematological parameters revealed that the addition of various concentrations of monoterpenes (MTs) to the diet had a significant impact on the physiological response of tilapia. There was a gradual decrease in mean corpuscular volume (MCV) and mean corpuscular haemoglobin (MCH), with the lowest values observed in fish receiving the 0.8 g kg^−1^ treatment. On the other hand, the mean corpuscular haemoglobin concentration (MCHC) was significantly higher (*p* < 0.05) in all fish treated with MTs and oxytetracycline (OTC) compared to the control group.

The highest red blood cell count (RBC) was observed in fish treated with 0.8 g kg^−1^ MTs, while the lowest count was found in fish treated with 0.4 g kg^−1^ MTs. Haematocrit (Ht) and haemoglobin (Hb) levels were significantly higher (*p* < 0.05) in the control group. Fish treated with 0.8 g kg^−1^ MTs showed a significant increase (*p* < 0.05) in total leucocyte count, as well as in lymphocytes, monocytes, eosinophils and basophils. Neutrophil count varied among groups but remained within normal levels for the species. Thrombocyte levels did not show significant differences (*p* > 0.05) between treatments, indicating stability in this parameter (Table 2).

**TABLE 2:** Haematological parameters (mean ± standard deviation) of juvenile Nile tilapia ( *O. niloticus* ) on the 10th day posttreatment for Edwardsiellosis with oxytetracycline (OTC) and varying concentrations of monoterpenes (MTs).

Parameters	Control	OTC	MTs 0.4 g kg^−1^	MTs 0.6 g kg^−1^	MTs 0.8 g	*p*
MCV (fL)	244.1 ± 63.0^c^	208.25 ± 38.50^bc^	220.7 ± 54.90^c^	178.35 ± 38.61^ab^	153.06 ± 27.44^a^	< 0.001
MCH (pg)	66.11 ± 17.18^bc^	71.03 ± 11.39^bc^	77.62 ± 18.19^c^	62.37 ± 13.45^ab^	51.56 ± 7.60^a^	< 0.001
MCHC (g dL^−1^)	26.98 ± 0.90^b^	34.38 ± 3.90^a^	35.09 ± 3.25^a^	35.01 ± 1.1^a^	34.01 ± 0.6^a^	< 0.001
RBC (× 10^6^ μL^−1^)	1.72 ± 0.47^ab^	1.33 ± 0.24^cd^	1.23 ± 0.24^d^	1.54 ± 0.29^bc^	1.93 ± 0.26^a^	< 0.001
Ht (%)	39.38 ± 2.02^a^	27.13 ± 4.39^b^	26.13 ± 1.65^b^	26.38 ± 0.75^b^	28.88 ± 2.53^b^	< 0.001
Hb (g.dL^−1^)	10.61 ± 0.39^a^	9.19 ± 0.34^c^	9.13 ± 0.42^c^	9.23 ± 0.36^c^	9.75 ± 0.39^b^	< 0.001
Total leucocytes (10^3^ μL^−1^)	61.63 ± 12.96^ab^	41.86 ± 5.60^b^	42.76 ± 10.18^b^	51.65 ± 21.63^b^	81.87 ± 3.98^a^	0.002
Lymphocytes (× 10^3^ μL^−1^)	48.62 ± 10.46^ab^	33.96 ± 4.54^b^	35.35 ± 8.44^b^	43.50 ± 18.24^b^	70.13 ± 3.40^a^	0.001
Monocytes (× 10^3^ μL^−1^)	2.96 ± 0.83^ab^	2.01 ± 1.9^bc^	1.74 ± 0.63^c^	2.20 ± 0.97^bc^	3.52 ± 0.48^a^	< 0.001
Neutrophil (× 10^3^ μL^−1^)	9.17 ± 1.90^a^	5.13 ± 0.66^b^	4.53 ± 1.20^b^	4.74 ± 2.01^b^	6.35 ± 0.29^ab^	0.001
Eosinophils (× 10^3^ μL^−1^)	1.43 ± 0.59^a^	0.83 ± 0.28^b^	0.80 ± 0.32^b^	0.86 ± 0.39^b^	1.39 ± 0.28^a^	< 0.001
Basophil (× 10^3^ μL^−1^)	2.13 ± 0.70^b^	2.26 ± 0.30^b^	2.15 ± 0.53^b^	2.2 ± 0.99^b^	3.48 ± 0.47^a^	0.040
Thrombocytes (× 10^3^ μL^−1^)	0.12 ± 0.22^a^	0.14 ± 0.19^a^	0.08 ± 0.19^a^	0.20 ± 0.30^a^	0.22 ± 0.35^a^	0.568

*Note:* Different letters (a,b,c) in the same line indicate statistically significant differences according to Tukey's test (*p* < 0.05).

Abbreviations: Hb, haemoglobin; Ht, haematocrit; MCH, mean corpuscular haemoglobin; MCHC, mean corpuscular haemoglobin concentration; MCV, mean corpuscular volume; RBC, red blood cell count.

### 3.3 Minimum Inhibitory Concentration

**FIGURE 2:**
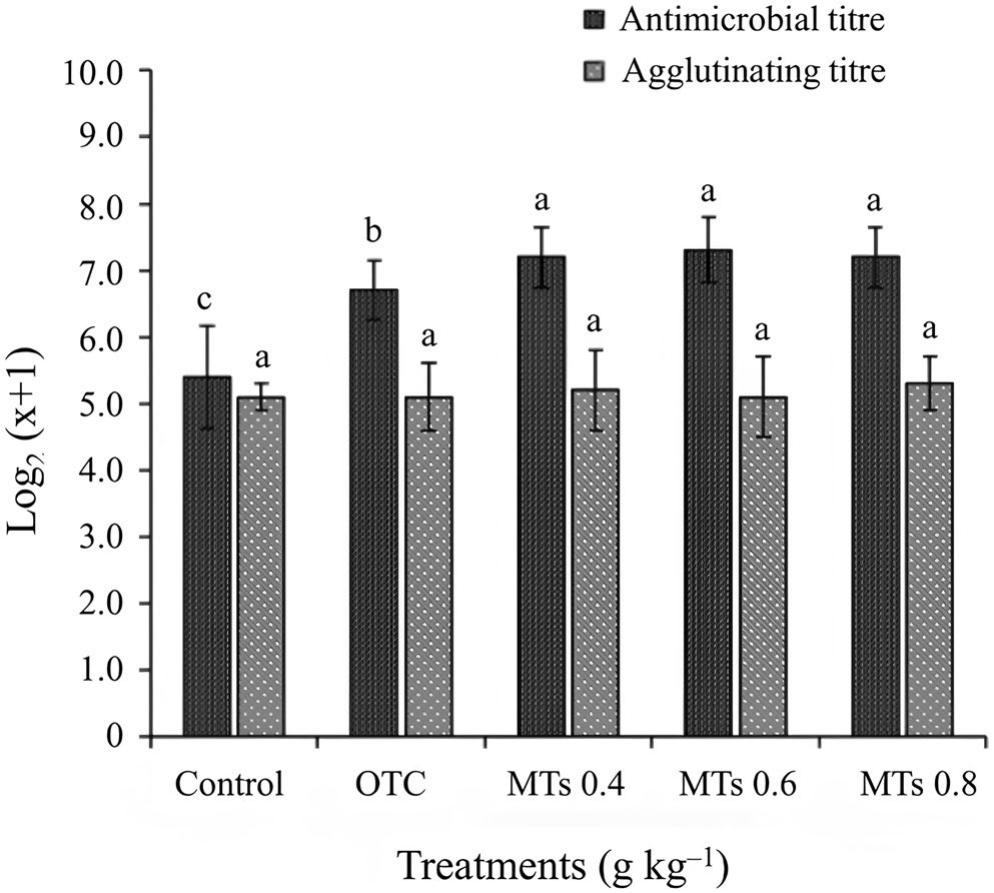
Antimicrobial titre and plasma agglutination of juvenile Nile tilapia ( *Oreochromis niloticus* ) after 10 days of treatment with: Control, OTC (oxytetracycline), MTs (monoterpenes): 0.4, 0.6 and 0.8 g kg^−1^ against *Edwardsiella tarda* . Different letters indicate significant differences according to Tukey's test (*p* < 0.05).

The results indicated significant differences (*p* < 0.05) in antimicrobial titre among the treatments. Fish in the control group showed a notable decrease in antimicrobial titre (*p* < 0.05) compared to fish treated with OTC and MTs. Fish treated with OTC had a significantly higher antimicrobial titre (*p* < 0.05) than the control group but lower (*p* < 0.05) than fish treated with MTs. Fish in the MT treatments did not show any significant differences between them. There were no statistically significant differences (*p* > 0.05) in the agglutinating titre parameter among the treatments (Figure 2).

## 4 Discussion

The present study assessed the effectiveness of combining thymol and carvacrol monoterpenes in treating juvenile Nile tilapia infected with *E tarda*. The results demonstrated that incorporating monoterpenes into fish feed led to a significant decrease in mortality rates, with a concentration of 0.8 g kg^−1^ proving to be as effective as oxytetracycline. The current health strategy outlined in the present study is in line with sustainability principles by advocating for a cleaner, safer and more efficient approach to treating fish diseases in aquaculture. Tavares‐Dias and Martins (2017) estimated that the total economic losses from parasites and infectious diseases amounted to US$84 million annually in Brazilian fish farming, excluding costs associated with medications and chemicals used for disease prevention and treatment.

While Oxytetracycline effectively reduced mortality to 3%, concerns about antimicrobial resistance have been raised due to its continued use (Liu et al. 2024; Manzoor et al. 2023). Therefore, natural alternatives like monoterpenes show promise. Although a lower dose of 0.4 g kg^−1^ was less effective, higher doses of monoterpenes have shown antimicrobial potential, possibly by boosting immune responses (Kachur and Suntres 2020), although excessive doses may lead to adverse effects (El‐Badawi et al. 2014). According to Caputo et al. (2023), aquaculture is frequently overlooked in the management of antimicrobial resistance (AMR). Antimicrobial chemotherapeutics are commonly used in aquaculture to prevent and treat bacterial infections (Preena et al. 2020). The persistent use of high concentrations of chemotherapeutic agents like oxytetracycline can result in the development of resistant bacterial strains. These agents can also accumulate in the sediment and water of aquaculture environments and in the muscles of fish meant for human consumption, posing a potential risk to public health (Owatari et al. 2024). Therefore, the substitution of antibiotics with natural products could offer numerous advantages to both human health and the aquaculture industry.

The application of monoterpenes (MTs) for treating juvenile Nile tilapia infected with *E. tarda* led to decreased mortality over the 10‐day treatment duration, demonstrating effectiveness similar to oxytetracycline (OTC), particularly at doses of 0.6 and 0.8 g kg^−1^. These findings are consistent with Nikolic et al. (2024), who reported synergistic antimicrobial effects of thymol and carvacrol when combined with antibiotics. *E. tarda* caused up to 53% mortality in untreated fish, and symptoms such as haemorrhages and organ enlargement were consistent with previous findings (Guo et al. 2022; Murwantoko et al. 2019; Preena et al. 2022).

The presence and efficacy of compounds like methyl salicylate, E‐anethole, thymol and carvacrol in animal feed are influenced by both the original formulation and the stability of these compounds during feed processing (EFSA et al. 2022). Research indicates that even if they are included in the initial formulation, volatile compounds such as methyl salicylate may not be detectable in the feed due to losses during processes like pelleting and drying, or limitations of the analytical methods used (Belhadj Slimen et al. 2025). Thymol and carvacrol are the most stable and easily detectable monoterpenes in the feed (EFSA et al. 2022; Gholami‐Ahangaran et al. 2022), and they are likely the main contributors to the therapeutic effects observed in the present study.

Over time, various new and recurring diseases have affected tilapia farming globally, leading to significant levels of illness and mortality, reduced production and trade limitations. Utilising effective management and disease prevention methods, such as the application of herbal remedies, can enhance the immunity of tilapia against these diseases (Wang et al. 2023). For example, adding Ginger ( *Z. officinale* ) essential oil to a diet at levels of 0.5%, 1.0% or 1.5% can boost phagocytic activity in Nile tilapia after 55 days of supplementation (Brum et al. 2017). Similarly, combining *Curcuma longa* hydrolate and *Lactobacillus plantarum* in the diet for 70 days improves the general health and survival of tilapia (Jatobá et al. 2024). Additionally, feeding experimental diets with 0%, 1% or 2% trub, a byproduct of the beer industry, for 50 days enhanced the innate immunity of tilapia, resulting in a low mortality rate of 2.08% in fish exposed to *Streptococcus agalactiae* (Gandolpho et al. 2025). However, most current nutritional approaches are used preventatively, as additives to ward off disease. In contrast, this study focused on using monoterpenes to treat existing illnesses. This showcases the innovative capabilities of the current strategy in using monoterpenes to treat diseases.

Comparative studies also support the efficacy of natural compounds *Withania somnifera* (0.7 g kg^−1^) and *Thymus vulgaris* (10 g kg^−1^) showed therapeutic potential (Mukherjee et al. 2022). Monoterpenes like thymol and carvacrol are linked to enhanced immune defences (Magouz et al. 2022; Singh et al. 2024; Valladão et al. 2019) and can replace antibiotics like OTC with fewer side effects (Abdallah et al. 2023; John 2024). Their selective mechanisms of action, such as not affecting agglutination titres, deserve further investigation (Iftikhar et al. 2021).

The findings of the current study also indicated that monoterpenes could lead to a decrease in MCV, potentially as a result of cytogenetic effects (Gomes et al. 2021; Valladão et al. 2019). MCH levels showed an increase at lower monoterpene doses but decreased at higher concentrations, indicating possible toxicity (Felix et al. 2023; Sadiqa et al. 2024). While MCHC levels increased in the treated groups, the differences were not statistically significant, with thymol and carvacrol possibly improving oxygen transport (Khalil et al. 2023; Yousefi et al. 2022).

The fish in the 0.8 g kg^−1^ MTs treatment showed higher RBC levels, potentially due to immunostimulatory effects (Ibrahim et al. 2022). However, Hb and Ht levels declined, indicating anaemia possibly caused by oxidative stress or toxicity (El‐Wafai et al. 2024; Orzuna‐Orzuna and Granados‐Rivera 2024).

Leucocyte counts increased in MT 0.8 g kg^−1^, especially lymphocytes, monocytes and basophils, suggesting activation of adaptive immunity, while neutrophils were lower likely due to tissue migration (Ibrahim et al. 2022; Valladão et al. 2019). High eosinophil and basophil counts indicated inflammation from infection (Costa et al. 2024; Ramadan et al. 2024). On the other hand, thrombocyte levels remained low across treatments, indicating limited effects of MTs on coagulation, although adequate thymol doses may increase their numbers (Frota et al. 2022; Naseer et al. 2024). Ultimately, monoterpenes proved effective in controlling *E. tarda* and offer a viable, residue‐free alternative to OTC (Paulino et al. 2022), though further studies on their molecular mechanisms and immune gene expression are needed (Felix et al. 2023; Silva et al. 2021). The enhanced activation of the cellular elements of the non‐specific immune response may account for the improved survival and recovery of fish infected with *E. tarda* . This is because the immune response involving defence cells like leucocytes fights off pathogens, halting their growth. Subsequently, the body initiates the repair of damaged cells, a process that can span from days to weeks, contingent on the infection's intensity.

Studies have shown that bacterial infections can lead to notable alterations in the expression of genes related to immune responses, including pro‐inflammatory cytokines (IL‐1β, IL‐6, TNF‐α), pathogen recognition receptors (NOD1, TLR‐7), antimicrobial proteins and components of the complement system. These changes have specific impacts on tissues like muscle, liver, spleen and kidney (Bakry et al. 2024; Saleh et al. 2024). Therefore, it is essential for future research to combine molecular and histopathological analyses to validate the proposed immunological mechanisms and assess potential effects on muscle tissue. This will help in developing more effective health management strategies and enhancing fish quality.

Monoterpenes show promise as they can help protect the environment and play a direct role in ensuring food safety and immunological protection. This study showed that incorporating the monoterpenes thymol and carvacrol into fish feed could be a promising approach for treating Nile tilapia infected with *E. tarda* . Sustainable feeding practices are crucial in contemporary fish farming to maintain productivity, animal well‐being and environmental conservation. Developing effective functional feeds could be a major breakthrough in the global aquaculture industry, particularly in managing *E. tarda* outbreaks.

## 5 Conclusion

The application of monoterpenes at levels of 0.6 and 0.8 g kg^−1^ proved to be successful in managing Edwardsiellosis and enhancing the immune system of Nile tilapia. Additionally, these levels showed similar effectiveness to oxytetracycline in reducing fish mortality. The findings suggest that monoterpenes could serve as a substitute for antibiotics in controlled settings. Future research should investigate residue accumulation in muscles, conduct field tests and assess their effectiveness against different bacterial strains.

## Author Contributions

Danilo Vitor Vilhena Batista: Formal Analysis, Writing – Original Draft, Visualisation; Adolfo Jatobá: Research, Methodology; Alexandre Vaz da Silva: Investigation, Methodology; Ana Paula de Souza: Investigation, Methodology; Marco Shizuo Owatari: Investigation, Methodology; Caio Francisco Santana Farias: Investigation, Methodology; Emilly Monteiro Lopes: Investigation, Methodology; Arlene Sobrinho Ventura: Formal Analysis, Methodology; Claudia Andrea Lima Cardoso: Formal Analysis, Methodology; Fernando Yugo Yamamoto: Research, Methodology, Writing – review and editing; Maurício Laterça Martins: Research, Project Administration, Funding; José Luiz Pedreira Mouriño: Advisor, Writing – review and editing, Project Administration.

## Ethics Statement

All the procedures carried out were in accordance with the rules of the National Council for the Control of Animal Experimentation, and the experimental protocol was approved by the Ethics Committee for the Use of Animals of the Federal Institute of Santa Catarina (CEUA/IFC) under No. 456/2024.

## Conflicts of Interest

The authors declare no conflicts of interest.

## Supporting information


**File S1.** Proximate composition of basal diet used to investigate the therapeutic efficacy of monoterpenes in Nile tilapia ( *Oreochromis niloticus* ) infected with *Edwardsiella tarda* . Information provided by the manufacturer.

## Data Availability

The data that support the findings of this study are available from the corresponding author upon reasonable request.
