# Supplementary material for: Therapeutic Efficacy of Monoterpenes in Nile Tilapia Infected With Edwardsiella tarda : A Phytogenic Alternative to Oxytetracycline
Source: J Fish Dis. 2025 Jul 31;49(2):e70032. doi: 10.1111/jfd.70032 (PMC12757894; doi:10.1111/jfd.70032)
Supplement: Supplementary file 1 — File S1. Proximate composition of basal diet used to investigate the therapeutic efficacy of monoterpenes in Nile tilapia ( Oreochromis niloticus ) infected with Edwardsiella tarda . Information provided by the manufacturer. [file JFD-49-e70032-s001.docx]

Supplementary file 1: Proximate composition of basal diet used to investigate the therapeutic efficacy of monoterpenes in Nile tilapia (*Oreochromis niloticus*) infected with *Edwardsiella tarda*. Information provided by the manufacturer.

| Ingredients | Content (%) |
| --- | --- |
| Crude Protein (min) | 46.0 |
| Moisture (max) | 12.0 |
| Ether Extract (min) | 8.0 |
| Fibrous Matter (max) | 3.0 |
| Mineral Matter (max) | 14.0 |
| Calcium (min-max) | 1.5~3.0 |
| Phosphorus (min) | 1.0 |
| Premix* | 2.0~4.0 |
| Digestible Energy (min) | 3,600 kcal/kg |

*Vitamin A (min) 20000IU/kg, Vitamin C (min) 750mg/kg, Vitamin D3 (min) 5000IU/kg, Vitamin E (min) 160IU/kg, Vitamin K3 (min) 10mg/kg, Vitamin B1 (min) 20mg/kg, Vitamin B2 (min) 25mg/kg, Vitamin B6 (min) 20mg/kg, Vitamin B12 (min) 135mcg/kg, Folic Acid (min) 8.5mg/kg, Pantothenic Acid (min) 100mg/kg, Biotin (min) 0.8mg/kg, Choline (min) 1800mg/kg, Niacin (min) 160mg/kg, Copper (min) 6mg/kg, Iron (min) 50mg/kg, Iodine (min) 1.3mg/kg, Manganese (min) 15mg/kg, Selenium (min) 0.3mg/kg, Zinc (min) 100mg/kg, Inositol (min) 250mg/kg.
